# Between‐country differences in the psychosocial profiles of British cattle farmers

**DOI:** 10.1002/vetr.5672

**Published:** 2025-10-12

**Authors:** Naomi S. Prosser, Eamonn Ferguson, Jasmeet Kaler, Edward M. Hill, Michael J. Tildesley, Matt J. Keeling, Martin J. Green

**Affiliations:** ^1^ School of Veterinary Medicine and Science University of Nottingham Sutton Bonington UK; ^2^ School of Psychology University of Nottingham Nottingham UK; ^3^ National Institute for Health and Care Research Blood and Transplant Research Unit in Donor Health and Behaviour University of Cambridge Cambridge UK; ^4^ Civic Health Innovation Labs and Institute of Population Health University of Liverpool Liverpool UK; ^5^ Zeeman Institute for Systems Biology and Infectious Disease Epidemiology Research University of Warwick Coventry UK; ^6^ Mathematics Institute and School of Life Sciences University of Warwick Coventry UK

**Keywords:** cattle, epidemiology, field trials, mastitis, statistics

## Abstract

**Background:**

Psychosocial factors are important for the uptake of livestock disease control measures by farmers and can differ by region, which would have implications for disease control nationally.

**Methods:**

We investigated altruism, trust, psychological proximity and the COM‐B behaviour change framework in a survey of 475 British cattle farmers in 2020. Using regression models, we studied associations between the country farmers lived in and psychosocial and behaviour change factors.

**Results:**

There were many between‐country differences in farmers' psychosocial and COM‐B profiles. Accounting for multiple tests, Scottish cattle farmers reported higher trust in governmental judgements for disease control and greater social opportunity to control disease than English cattle farmers.

**Limitations:**

There were relatively low numbers of respondents from Scotland and Wales. As such, the results should be interpreted with caution. Northern Irish farmers could not be included in the analyses as there were too few responses.

**Conclusion:**

Cattle farmers differed in their psychosocial profiles by country. Our sample of Scottish farmers reported higher trust in, and felt better supported by, government in the context of disease control than the English farmers, which could be due to different disease control approaches between devolved governments. Understanding between‐country differences in farmer psychosocial attributes has implications for animal health governance and approaches to disease control.

## INTRODUCTION

Numerous endemic diseases limit the productivity of cattle herds and incur costs for prevention and treatment.^1^ Disease outbreaks and control strategies are also very costly to the industry.^2^ Livestock disease control involves multiple actors and differs depending on the disease. Most disease control is farmer‐led, with farmers making decisions within their own herds, often with input from veterinarians. However, some diseases are also regulated by the government, such as bovine tuberculosis,^3^ and others that are under voluntary control may have industry‐led schemes, such as bovine viral diarrhoea.^4^

Psychosocial factors are important for disease control both on individual farms and nationally. However, these can work in diverse and not necessarily synergistic ways. For example, one study showed that farmers with low trust in other farmers were more likely to control bovine viral diarrhoea,^5^ perhaps to protect their herd from the disease risk posed by herds managed by farmers they did not trust to control disease. On the other hand, trust in others is commonly identified as important for farmer uptake of livestock disease control, with high trust required for cooperative action for regional and national control and eradication of diseases such as bovine viral diarrhoea.^6^, ^7^ It has been reported that veterinary advice is generally trusted by farmers,^5^, ^8^ while trust in governmental judgements for livestock disease control can be associated with earlier intervention to prevent disease.^9^ Psychological proximity, which is how close a person feels to another person (in terms of connection, independence, behavioural closeness and similarities),^10^ is another important factor, and it has been reported that farmers with high psychological proximity to their veterinarian are more likely to use many measures to control bovine viral diarrhoea.^5^

In addition to psychosocial factors, the COM‐B framework of behaviour change theorises capability (physical and psychological), opportunity (physical and social) and motivation (automatic and reflective) being interrelated influences of behaviour.^11^, ^12^ Physical capability is the physical capacity and skills to do an activity (i.e., the farmer is physically able to control disease), and psychological capability is having sufficient knowledge and understanding of how and why to do a behaviour (i.e., the farmer knows how and why to control disease). Physical opportunity is having the necessary infrastructure (i.e., farm facilities to control disease), and social opportunity is having sufficient support from people to allow a behaviour (i.e., support from other members of the farm team to control disease). Finally, automatic motivation is the habitual and emotional decision making for a behaviour, while reflective motivation reflects the conscious goals influencing decision making. The framework was developed in human health behaviour and has recently been applied to livestock disease control by farmers, with all factors (capability, opportunity and motivation) identified as important in farmer uptake of different disease control measures.^5^, ^9^, ^13^

Farmer behaviour is known to vary, and this has implications for disease control in livestock, which is often farmer‐led.^5^, ^9^, ^14^ Psychosocial factors can vary between cultures and regions. For example, there are national differences in altruism and prosociality,^15^, ^16^, ^17^ and in trust in people^18^ and institutions^19^ in general. These differences can also be found between areas within the same country. For example, in China, there are reported regional differences in trust in others and the impact that close relationships have on behaviour,^20^ and within the UK, there are regional differences in trust in government.^21^, ^22^

Regional differences in factors associated with livestock disease control will have implications for the design and success of national disease intervention strategies because interventions may have different effects between regions. Therefore, the aim of this research was to evaluate between‐country differences in the psychosocial and behavioural characteristics of British cattle farmers.

## MATERIALS AND METHODS

### Survey data

We used data from a previously conducted survey completed by 475 UK cattle farmers in 2020. Ethical approval was obtained from the University of Nottingham Research Ethics Committee (reference number: 2789 190711, granted on 22 June 2019). The purpose of the survey was to investigate psychosocial and COM‐B factors in UK farmers and how they were associated with disease control. A detailed description of the survey and data collected has been reported previously.^5^ In brief, the survey was distributed to UK cattle farmers and investigated altruism, trust in others and psychological proximity to others using multiple validated scales.^10^, ^23^, ^24^ Altruism was measured using the social value orientation slider measure, which scored farmers on a scale from competitive to altruistic.^23^ Trust was measured using a series of Likert‐scale statements about trust and distrust in other farmers, veterinarians, the National Farmers Union (NFU) and government, both in general and in the context of infectious disease control.^25^ These were converted to a numeric scale (1 = strongly disagree, 2 = disagree, 3 = neither agree nor disagree, 4 = agree and 5 = strongly agree). We investigated the psychological proximity the farmers felt to their cows, other farmers, veterinarians, the NFU and government using the ‘inclusion of other in the self’ scale, which scored farmers for how close they felt to the ‘other’.^10^, ^24^ Finally, we assessed the COM‐B factors using a series of Likert‐scale statements that investigated each aspect of the COM‐B framework for farmers to control infectious diseases in their cattle in general. These were converted to numeric scores in the same way as for the trust questions, and we computed mean scores for a set of statements within each COM‐B factor.

Of the 475 farmers who completed the survey, there were 19 that did not provide the country they farmed within, and only 10 from Northern Ireland, so we omitted these, leaving the responses from 446 farmers for the analysis. There were 342 farmers from England, 69 farmers from Scotland and 35 farmers from Wales. Some questions had missing responses; a summary of the number of missing responses per variable of interest is provided in Table S1. We were not able to calculate a response rate because the survey was advertised using diverse methods, including online, so we do not know how many farmers saw the survey.

### Psychosocial and COM‐B factor regression models

All data analysis was conducted in R (version 4.2.2).^26^ We investigated associations between farm location (country) and farmer psychosocial and behaviour change factors using univariable linear regression models created with the lme4 R package.^27^ We also included farmer age (under 40 years, 40‒49 years, 50‒59 years and >60 years) and herd type (beef or dairy) in the models to control for possible confounding. The number of farmers from each country by herd type and age is in Table S2. The models took the form:

$$y\;∼\;\beta_{0}+\sum_{i=1}^{p}\beta_{i}x_{i}+e$$

where *y* is a psychosocial or COM‐B factor, *∼* is an identity link, *β*_0_ is the intercept, *β_i_* are coefficients for *i‒p* variables *x_i_* (country, farmer age and herd type) and *e* is the residual model error.

We applied a Bonferroni correction factor to a significance threshold of 0.05 to account for the repeated testing (34 factors investigated, Table S3), which resulted in a corrected significance threshold of 0.0015. However, the Bonferroni correction is conservative when there are many tests and when the tests are of correlated variables^28^, ^29^; both conditions were the case here. We therefore also report models that met a 0.05 significance threshold.

### Data reduction using *k*‐mean clustering

We also undertook a data‐reduction approach using *k*‐means^30^ to identify clusters of farmers based on their broader psychosocial and COM‐B factor profiles. For this analysis, we included those farmers who gave responses to all factors used in the clustering (a total of 389 farmers). We selected the number of clusters that resulted in the greatest reduction in the within‐cluster sum of squares compared to one fewer cluster.^31^ Associations between the resulting clusters and country were tested using a logistic regression model.

## RESULTS

### Between‐country differences in psychosocial and COM‐B factors

When the Bonferroni‐corrected *p*‐value threshold of 0.0015 was used, we found that two psychosocial and COM‐B variables were different between cattle farmers in different countries. The Scottish cattle farmers had higher trust in governmental judgements for disease control (coefficient = 0.51, *p* < 0.001; Table 1) and had greater social opportunity for controlling disease (questions covered enabling support and encouragement from other farmers, veterinarians and government policy) (coefficient = 0.21, *p* = 0.001; Table 2) than the English cattle farmers.

**TABLE 1:** Coefficients, 95% confidence intervals and *p*‐values from a linear regression model of farmer trust in governmental judgements for infectious disease control (on a scale of 1 [low]–5 [high]), with country as the explanatory variable.

Model and variable	Coefficient	95% confidence interval	*p*‐Value
Country‐level model (reference = Scotland)			
Intercept	2.83	2.60‒3.06	
England	‒0.51	‒0.76 to ‒0.25	<0.001
Wales	‒0.36	‒0.76 to 0.05	0.083

*Note*: Neither herd type nor farmer age was a confounder.

**TABLE 2:** Coefficients, 95% confidence intervals and *p*‐values from a linear regression model of farmer social opportunity for infectious disease control (on a scale of 1 [low]–5 [high]), with country as the explanatory variable.

Model and variable	Coefficient	95% confidence interval	*p*‐Value
Country‐level model (reference = Scotland)			
Intercept	3.57	3.46‒3.68	
England	‒0.21	‒0.33 to ‒0.09	0.001
Wales	‒0.22	‒0.41 to ‒0.03	0.022

*Note*: Neither herd type nor farmer age was a confounder.

**FIGURE 1:**
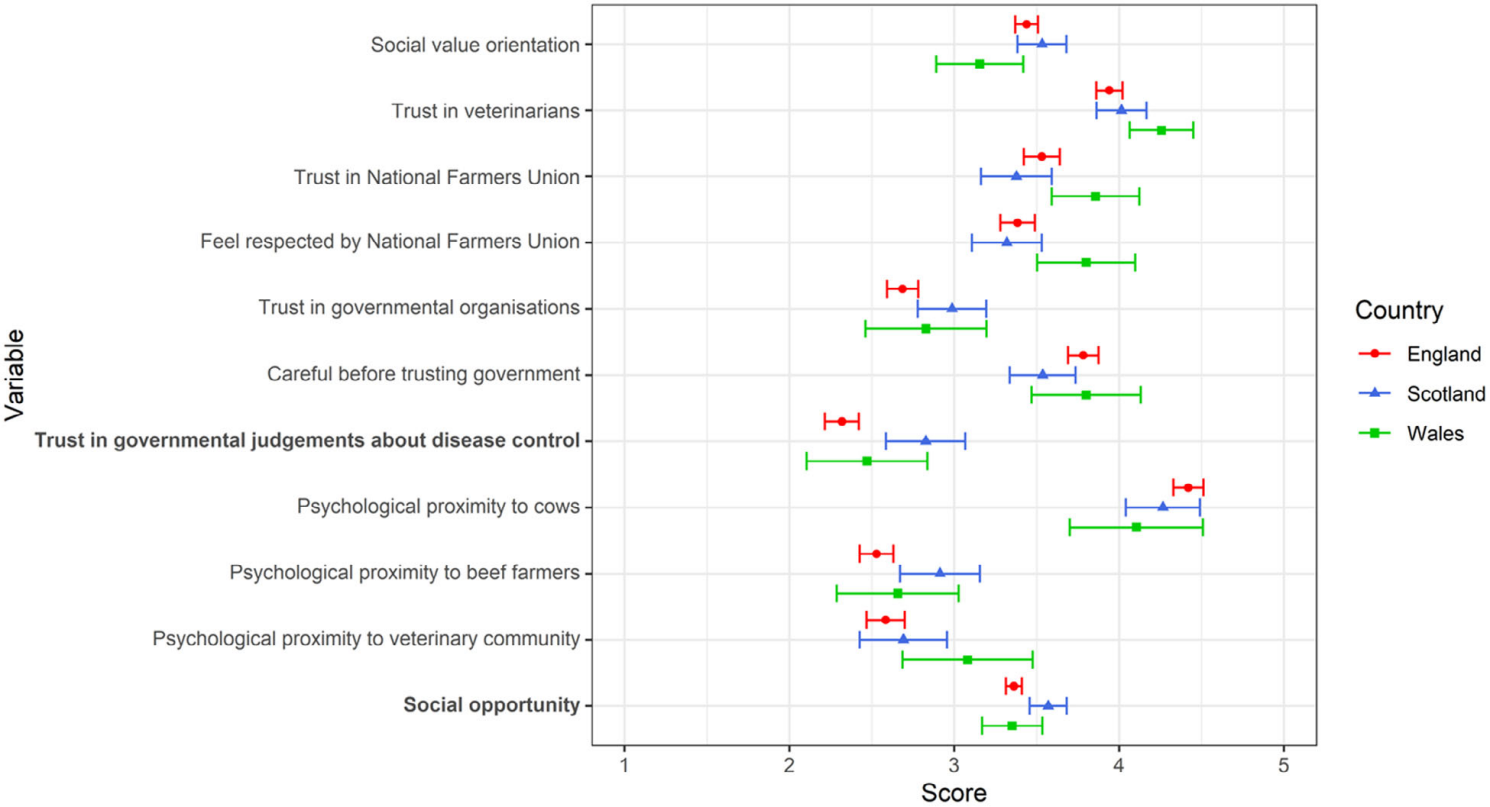
Means and 95% confidence intervals (adjusted to a 1 [low]–5 [high] scale) of the 11 psychosocial and COM‐B factors that were different between at least two countries at a significance level of 0.05. Variables in bold font were also different at a significance level of 0.0015. Neither herd type nor farmer age was a confounder. England is denoted by a red circle, Scotland by a blue triangle and Wales by a green square. A high score for social value orientation indicates high altruism.

When a less conservative *p*‐value threshold of 0.05 was used, there were multiple differences in psychosocial and behavioural factors between countries (Figure 1 and Table S3). The Scottish cattle farmers were more altruistic to other cattle farmers than the Welsh cattle farmers (coefficient = 7.00, *p* = 0.011) and, compared to the English cattle farmers, had higher psychological proximity to beef farmers (coefficient = 0.45, *p* = 0.019) and higher trust in government (coefficient = 0.31, *p* = 0.009). Compared to the English cattle farmers, the Welsh cattle farmers had higher trust in veterinarians (coefficient = 0.34, *p* = 0.008) and felt closer to the veterinary community (coefficient = 0.68, *p* = 0.023). Compared to the Scottish cattle farmers, the Welsh cattle farmers had higher trust in the NFU (coefficient = 0.45, *p* = 0.032). The Welsh cattle farmers also felt more respected by the NFU than both the English (coefficient = 0.39, *p* = 0.022) and the Scottish (coefficient = 0.46, *p* = 0.023) cattle farmers. Finally, the English cattle farmers had higher psychological proximity to their cows than the Welsh farmers (coefficient = 0.48, *p* = 0.045).

Post hoc power analysis (using the pwr R package^32^) was conducted to investigate the effect sizes that could be detected at both the 0.0015 and 0.05 significance levels at 80% power between the countries with the smallest samples of farmers (Wales and Scotland), using the standard deviation of the total sample of cattle farmers for each variable in the calculation. For a significance level of 0.0015, the effect sizes that could be detected at 80% power were 11.15 for social value orientation, 0.61–0.86 for the trust variables, 1.06–1.46 for the psychological proximity variables and 0.40–0.67 for the COM‐B variables (Table S4). For a significance level of 0.05, this was reduced to effect sizes of 7.65 for social value orientation, 0.42–0.59 for the trust variables, 0.73–1.00 for the psychological proximity variables and 0.27–0.46 for the COM‐B variables (Table S5).

### Country differences in psychosocial and COM‐B clusters

The *k*‐means clustering of farmers by the psychosocial and COM‐B factors resulted in two clusters as the best fit (Figure S1). Farmers in cluster one had higher scores across all psychosocial and COM‐B factors than farmers in cluster two (Table 3). In the binomial logistic regression model of country associated with psychosocial/COM‐B cluster, the English farmers had an increased likelihood of being in the low scoring cluster (cluster 2) compared with the Welsh farmers (odds ratio = 3.03; Table 4).

**TABLE 3:** Mean psychosocial and COM‐B scores of cattle farmers (*n* = 389) in the two clusters resulting from *k*‐means clustering of the factors.

Factor		Cluster 1	Cluster 2
Altruism	Social value orientation	32.54	29.93
Trust	Trust in beef farmers	3.52	3.00
	Trust in dairy farmers	3.69	3.24
	Careful before trusting farmers	3.29	3.61
	Trust farmers met for first time	3.07	2.66
	Trust neighbours to control disease	3.24	2.70
	Trust farmers to control disease	3.10	2.74
	Trust veterinarians	4.35	3.62
	Feel respected by veterinarian	4.49	3.79
	Feel respected by veterinarians	4.20	3.24
	Careful before trust veterinarians	2.51	3.17
	Trust veterinary advice about disease control	4.67	4.02
	Farmers receive high‐quality veterinary advice	4.34	3.57
	Veterinarian would always tell truth	4.69	3.96
	Trust National Farmers Union	3.98	3.06
	Feel respected by National Farmers Union	3.83	2.95
	Trust governmental organisations	3.13	2.39
	Feels respected by government	2.65	2.05
	Careful before trust government	3.56	3.94
	Trust governmental judgements about disease control	2.79	2.05
Psychological proximity	Psychological proximity to cows	6.34	5.81
	Psychological proximity to beef farmers	4.08	2.83
	Psychological proximity to dairy farmers	4.02	2.70
	Psychological proximity to neighbouring farmers	4.56	3.33
	Psychological proximity to farming community	3.99	2.74
	Psychological proximity to veterinarian	5.72	4.05
	Psychological proximity to veterinary community	4.32	2.66
	Psychological proximity to National Farmers Union	3.69	2.28
	Psychological proximity to government	2.38	1.57
COM‐B	Psychological capability	4.40	4.11
	Physical opportunity	4.15	3.80
	Social opportunity	3.63	3.17
	Automatic motivation	4.42	4.16
	Reflective motivation	4.56	4.27

**TABLE 4:** Odds ratios, 95% confidence intervals and *p*‐values from a logistic regression model of the outcome of cattle farmers being in psychosocial and COM‐B cluster 2 (low scoring cluster) compared to cluster 1 (high scoring cluster), with country as the explanatory variable.

Model and variable	Odds ratio	95% confidence interval	*p*‐Value
Country‐level model (reference = Wales)			
England	3.03	1.33‒7.56	0.011
Scotland	1.98	0.78‒5.39	0.162

*Note*: Neither herd type nor farmer age was a confounder.

## DISCUSSION

This study aimed to investigate regional differences in psychosocial and COM‐B factors in UK cattle farmers that may have implications for successful disease control. There were differences in these factors between specific countries of the UK in our sample of farmers.

A notable finding from this research was that the Scottish cattle farmers had higher trust in governmental decisions for disease control than the English cattle farmers. Livestock disease is a devolved issue in the UK^33^; therefore, the experiences that farmers have of legislation and governance of cattle disease will differ depending on the country in which they farm. For example, the devolved governments have different approaches to bovine viral diarrhoea control. In Scotland, a control scheme with the aim of eradication was co‐produced between government and farmers^34^ and testing is mandatory,^35^ while England has had a voluntary industry‐led scheme that has not managed to engage the majority of farmers.^4^, ^36^ There are also different cattle disease risks between Scotland and England. Scotland has a very low incidence of bovine tuberculosis, with very few herd breakdowns resulting in infrequent herd testing. In contrast, England has a diverse prevalence of bovine tuberculosis across its regions, with many areas suffering frequent herd breakdowns and, as a result, experiencing more frequent mandatory testing in negative herds.^37^ Bovine tuberculosis is a highly political issue, and farmers from high‐risk areas generally have a lack of trust in government for controlling bovine tuberculosis.^38^, ^39^ The higher trust the Scottish cattle farmers had in government is similar to that felt by the general Scottish population, who have relatively high trust in the Scottish government and low trust in the UK government.^21^, ^22^ In general, a lack of trust in government by farmers can result from a lack of long‐term and consistent contact with government^40^; it has been reported that stakeholders in England feel that agriculture is a lower priority for the English government than it is for the Scottish and Welsh governments.^41^ Therefore, addressing these issues could result in cattle farmers having increased trust in the government. However, we did not investigate how the trust in governmental judgements differed for diseases that may be governed in other ways and what implications this may have on farmer behaviour for disease control.

The Scottish cattle farmers had higher social opportunity to control disease than the English cattle farmers. Our questions on social opportunity to control disease covered a range of relevant people and groups that may influence behaviour, including support from government (Supporting Information S1). Therefore, the perceived increased support that Scottish cattle farmers receive is likely to influence their increased trust, but also includes support from others, such as veterinarians and other farmers. The lower perceived support from government, veterinarians and other farmers in England indicates that increasing social opportunity provided by these groups of people may be important to improve disease control by cattle farmers. Accordingly, it is important to identify specific groups that may be limiting the social opportunity of cattle farmers to control disease among their herds. Such identification would enable the design of an intervention to improve social opportunity for English cattle farmers. The different disease experiences of each nation could also be influencing the farmers' social opportunity to control disease; for example, being bovine tuberculosis‐free gives Scotland extra resources for controlling other diseases, such as bovine viral diarrhoea.^34^ Social opportunity is commonly identified as important in farmer behaviour for cattle disease control,^42^ but finding a between‐country difference in social opportunity among British cattle farmers is novel.

The Welsh cattle farmers had higher trust in and psychological proximity to veterinarians than the English cattle farmers, higher trust in the NFU than the Scottish cattle farmers, and generally had higher scores for many of the psychosocial and COM‐B factors than the English cattle farmers. Veterinarians are a trusted source of advice for farmers.^5^, ^8^, ^43^ However, research generally combines farmers from the four UK nations, which masks any differences between the countries. It was notable that, in all countries, there was a relatively high trust in veterinarians and low trust in governments. The higher trust in the NFU by the Welsh cattle farmers compared to the other nations is an interesting result since, in the past, NFU membership in Wales was proportionally lower than in England,^44^ and Welsh farmers also had the choice of joining an alternative union (Farmers’ Union of Wales).

This study is limited by the small number of survey participants from all nations except England and the disparity in the number of participants from each country, which means that the results should be interpreted with caution. Thirty‐five and 69 farmers are not enough to be representative of all farms in Wales and Scotland, respectively, and we do not know how representative these samples are. Therefore, although we have found some interesting and novel differences in some psychosocial factors important for disease control in this sample of farmers, further study with a larger and more representative sample of farmers from each nation is needed to confirm these findings. The farmers were recruited through diverse cattle‐interest organisations, some of which had a regional reach, which may have influenced the sample of cattle farmers recruited to the survey from each region. We also have not investigated whether the identified differences in trust and social opportunity translate into behavioural differences in disease control between the farmers from each country, and this would be an important area of future research.

## CONCLUSION

In conclusion, in our sample of cattle farmers, the Scottish farmers had higher trust in governmental judgements for disease control and higher social opportunity than the English farmers. One explanation for this could be the devolved government in Scotland and their differing approach to livestock disease control compared to England. However, care needs to be taken in interpreting such a small sample of farmers and an unbalanced dataset, which may not be representative. The differences found in our sample of cattle farmers may have implications regarding the risk of diseases affecting cattle farms and should be considered when developing cattle farm disease control approaches in the future.

## AUTHOR CONTRIBUTIONS

Martin Green, Michael Tildesley, Jasmeet Kaler, Eamonn Ferguson and Matt Keeling were responsible for funding acquisition and supervision. Naomi Prosser, Martin Green, Eamonn Ferguson and Jasmeet Kaler were involved in the conceptualisation, formal analysis, investigation and methodology. Naomi Prosser curated the data and wrote the original draft. All the authors contributed to the review and editing of the paper.

## CONFLICT OF INTEREST STATEMENT

The authors declare they have no conflicts of interest.

## ETHICS STATEMENT

Ethical approval was obtained from the University of Nottingham Research Ethics Committee (reference number: 2789 190711, granted on 22 June 2019).

## Supporting information



Supporting Information

Supporting Information

## Data Availability

The data analysis code is available as Supporting Information. Research data are not shared as we did not obtain participants' consent to share the survey responses.
