# Supplementary material for: Between‐country differences in the psychosocial profiles of British cattle farmers
Source: Vet Rec. 2025 Oct 12;198(4):e159–65. doi: 10.1002/vetr.5672 (PMC12904086; doi:10.1002/vetr.5672)
Supplement: Supplementary file 1 — Supporting Information [file VETR-198--s001.docx]

**Supplementary Information**

*Supplementary Table 1. The number of missing responses for each psychosocial and COM-B factor for 446 British cattle farmers.*

| Factor | | Number of missing responses |
| --- | --- | --- |
| Altruism | Social value orientation | 26 |
| Trust | Trust in beef farmers | 9 |
|  | Trust in dairy farmers | 8 |
|  | Careful before trusting farmers | 7 |
|  | Trust farmers met for first time | 7 |
|  | Trust neighbours to control disease | 7 |
|  | Trust farmers to control disease | 5 |
|  | Trust veterinarians | 5 |
|  | Feel respected by veterinarian | 3 |
|  | Feel respected by veterinarians | 5 |
|  | Careful before trust veterinarians | 6 |
|  | Trust veterinary advice about disease control | 6 |
|  | Farmers receive high quality veterinary advice | 6 |
|  | Veterinarian would always tell truth | 5 |
|  | Trust National Farmers Union | 5 |
|  | Feel respected by National Farmers Union | 6 |
|  | Trust governmental organisations | 7 |
|  | Feels respected by government | 6 |
|  | Careful before trust government | 6 |
|  | Trust governmental judgements about disease control | 7 |
| Psychological proximity | Psychological proximity to cows | 12 |
|  | Psychological proximity to beef farmers | 11 |
|  | Psychological proximity to dairy farmers | 13 |
|  | Psychological proximity to neighbouring farmers | 7 |
|  | Psychological proximity to farming community | 11 |
|  | Psychological proximity to veterinarian | 11 |
|  | Psychological proximity to veterinary community | 11 |
|  | Psychological proximity to National Farmers Union | 9 |
|  | Psychological proximity to government | 9 |
| COM-B | Psychological capability | 5 |
|  | Physical opportunity | 4 |
|  | Social opportunity | 3 |
|  | Automatic motivation | 5 |
|  | Reflective motivation | 5 |

*Supplementary Table 2. The coefficients and 95% confidence intervals from linear regression models of farmer country, farmer age and herd type associated with psychosocial and COM-B behaviour change factors for 446 British cattle farmers, with reference categories of Wales (Country), Beef (Herd type) and Under 40 (Age).*

| Model | Variable | Coefficient | 95% confidence interval | P-value |
| --- | --- | --- | --- | --- |
| Social value orientation | Intercept | 26.89 | 21.52 - 32.25 |  |
| Social value orientation | Country: England | 5.30 | 0.63 - 9.96 | 0.027 |
| Social value orientation | Country: Scotland | 7.00 | 1.60 - 12.40 | 0.011 |
| Social value orientation | Herd type: Dairy | -1.08 | -3.59 - 1.42 | 0.397 |
| Social value orientation | Age: 40-49 | -0.96 | -4.95 - 3.03 | 0.638 |
| Social value orientation | Age: 50-59 | -1.20 | -4.70 - 2.30 | 0.502 |
| Social value orientation | Age: Over 60 | -0.44 | -3.90 - 3.01 | 0.801 |
| Trust beef farmers | Intercept | 3.50 | 3.18 - 3.81 |  |
| Trust beef farmers | Country: England | 0.04 | -0.23 - 0.31 | 0.781 |
| Trust beef farmers | Country: Scotland | -0.04 | -0.36 - 0.27 | 0.795 |
| Trust beef farmers | Herd type: Dairy | -0.36 | -0.51 - -0.21 | < 0.001 |
| Trust beef farmers | Age: 40-49 | -0.19 | -0.44 - 0.05 | 0.122 |
| Trust beef farmers | Age: 50-59 | -0.13 | -0.34 - 0.09 | 0.238 |
| Trust beef farmers | Age: Over 60 | -0.07 | -0.28 - 0.14 | 0.501 |
| Trust dairy farmers | Intercept | 3.61 | 3.29 - 3.92 |  |
| Trust dairy farmers | Country: England | -0.07 | -0.34 - 0.20 | 0.629 |
| Trust dairy farmers | Country: Scotland | -0.19 | -0.51 - 0.13 | 0.240 |
| Trust dairy farmers | Herd type: Dairy | 0.08 | -0.07 - 0.23 | 0.296 |
| Trust dairy farmers | Age: 40-49 | 0.09 | -0.15 - 0.33 | 0.464 |
| Trust dairy farmers | Age: 50-59 | -0.15 | -0.37 - 0.06 | 0.155 |
| Trust dairy farmers | Age: Over 60 | -0.18 | -0.39 - 0.03 | 0.093 |
| Trust farmers met for first time | Intercept | 2.98 | 2.67 - 3.30 |  |
| Trust farmers met for first time | Country: England | -0.02 | -0.28 - 0.25 | 0.892 |
| Trust farmers met for first time | Country: Scotland | 0.00 | -0.31 - 0.32 | 0.987 |
| Trust farmers met for first time | Herd type: Dairy | -0.17 | -0.32 - -0.02 | 0.024 |
| Trust farmers met for first time | Age: 40-49 | -0.07 | -0.32 - 0.17 | 0.550 |
| Trust farmers met for first time | Age: 50-59 | -0.03 | -0.24 - 0.18 | 0.765 |
| Trust farmers met for first time | Age: Over 60 | -0.05 | -0.26 - 0.15 | 0.611 |
| Careful before trust farmers | Intercept | 3.28 | 2.92 - 3.64 |  |
| Careful before trust farmers | Country: England | 0.11 | -0.19 - 0.42 | 0.470 |
| Careful before trust farmers | Country: Scotland | 0.12 | -0.24 - 0.47 | 0.526 |
| Careful before trust farmers | Herd type: Dairy | 0.10 | -0.07 - 0.27 | 0.235 |
| Careful before trust farmers | Age: 40-49 | -0.08 | -0.35 - 0.20 | 0.589 |
| Careful before trust farmers | Age: 50-59 | -0.06 | -0.30 - 0.18 | 0.640 |
| Careful before trust farmers | Age: Over 60 | 0.24 | 0.01 - 0.48 | 0.045 |
| Trust neighbours to control disease | Intercept | 3.04 | 2.63 - 3.45 |  |
| Trust neighbours to control disease | Country: England | -0.22 | -0.57 - 0.13 | 0.212 |
| Trust neighbours to control disease | Country: Scotland | -0.23 | -0.64 - 0.18 | 0.275 |
| Trust neighbours to control disease | Herd type: Dairy | 0.02 | -0.18 - 0.21 | 0.866 |
| Trust neighbours to control disease | Age: 40-49 | 0.24 | -0.07 - 0.55 | 0.136 |
| Trust neighbours to control disease | Age: 50-59 | 0.14 | -0.13 - 0.42 | 0.314 |
| Trust neighbours to control disease | Age: Over 60 | 0.18 | -0.09 - 0.45 | 0.193 |
| Trust farmers to control disease | Intercept | 3.04 | 2.67 - 3.41 |  |
| Trust farmers to control disease | Country: England | -0.18 | -0.49 - 0.13 | 0.261 |
| Trust farmers to control disease | Country: Scotland | -0.27 | -0.64 - 0.10 | 0.147 |
| Trust farmers to control disease | Herd type: Dairy | 0.04 | -0.14 - 0.22 | 0.651 |
| Trust farmers to control disease | Age: 40-49 | 0.12 | -0.17 - 0.41 | 0.411 |
| Trust farmers to control disease | Age: 50-59 | -0.02 | -0.27 - 0.23 | 0.882 |
| Trust farmers to control disease | Age: Over 60 | 0.15 | -0.09 - 0.40 | 0.219 |
| Trust vets | Intercept | 4.39 | 4.10 - 4.69 |  |
| Trust vets | Country: England | -0.34 | -0.59 - -0.09 | 0.008 |
| Trust vets | Country: Scotland | -0.29 | -0.58 - 0.01 | 0.058 |
| Trust vets | Herd type: Dairy | -0.14 | -0.28 - 0.00 | 0.050 |
| Trust vets | Age: 40-49 | -0.09 | -0.32 - 0.14 | 0.424 |
| Trust vets | Age: 50-59 | -0.14 | -0.34 - 0.06 | 0.186 |
| Trust vets | Age: Over 60 | 0.01 | -0.18 - 0.21 | 0.891 |
| Feels respected by vet | Intercept | 4.22 | 3.91 - 4.52 |  |
| Feels respected by vet | Country: England | -0.12 | -0.38 - 0.14 | 0.375 |
| Feels respected by vet | Country: Scotland | -0.08 | -0.38 - 0.23 | 0.631 |
| Feels respected by vet | Herd type: Dairy | 0.14 | 0.00 - 0.29 | 0.055 |
| Feels respected by vet | Age: 40-49 | -0.11 | -0.35 - 0.13 | 0.365 |
| Feels respected by vet | Age: 50-59 | -0.14 | -0.35 - 0.06 | 0.174 |
| Feels respected by vet | Age: Over 60 | 0.07 | -0.13 - 0.27 | 0.506 |
| Feels respected by vets | Intercept | 3.85 | 3.50 - 4.20 |  |
| Feels respected by vets | Country: England | -0.05 | -0.35 - 0.24 | 0.724 |
| Feels respected by vets | Country: Scotland | 0.07 | -0.28 - 0.42 | 0.677 |
| Feels respected by vets | Herd type: Dairy | -0.14 | -0.31 - 0.02 | 0.093 |
| Feels respected by vets | Age: 40-49 | -0.17 | -0.44 - 0.10 | 0.226 |
| Feels respected by vets | Age: 50-59 | -0.13 | -0.37 - 0.10 | 0.266 |
| Feels respected by vets | Age: Over 60 | 0.03 | -0.20 - 0.26 | 0.783 |
| Careful before trust vets | Intercept | 2.72 | 2.30 - 3.14 |  |
| Careful before trust vets | Country: England | 0.09 | -0.26 - 0.44 | 0.622 |
| Careful before trust vets | Country: Scotland | -0.15 | -0.57 - 0.27 | 0.479 |
| Careful before trust vets | Herd type: Dairy | 0.21 | 0.01 - 0.40 | 0.041 |
| Careful before trust vets | Age: 40-49 | 0.13 | -0.19 - 0.46 | 0.413 |
| Careful before trust vets | Age: 50-59 | -0.12 | -0.40 - 0.17 | 0.420 |
| Careful before trust vets | Age: Over 60 | 0.09 | -0.18 - 0.37 | 0.506 |
| Vet would always tell truth | Intercept | 4.48 | 4.18 - 4.77 |  |
| Vet would always tell truth | Country: England | -0.16 | -0.41 - 0.09 | 0.208 |
| Vet would always tell truth | Country: Scotland | -0.10 | -0.39 - 0.20 | 0.527 |
| Vet would always tell truth | Herd type: Dairy | -0.05 | -0.19 - 0.09 | 0.468 |
| Vet would always tell truth | Age: 40-49 | 0.01 | -0.22 - 0.24 | 0.934 |
| Vet would always tell truth | Age: 50-59 | -0.09 | -0.29 - 0.11 | 0.366 |
| Vet would always tell truth | Age: Over 60 | 0.09 | -0.11 - 0.28 | 0.393 |
| Trust vet advice about disease control | Intercept | 4.38 | 4.07 - 4.69 |  |
| Trust vet advice about disease control | Country: England | -0.08 | -0.34 - 0.18 | 0.545 |
| Trust vet advice about disease control | Country: Scotland | 0.01 | -0.30 - 0.32 | 0.942 |
| Trust vet advice about disease control | Herd type: Dairy | 0.01 | -0.14 - 0.16 | 0.888 |
| Trust vet advice about disease control | Age: 40-49 | 0.02 | -0.22 - 0.26 | 0.854 |
| Trust vet advice about disease control | Age: 50-59 | -0.04 | -0.25 - 0.17 | 0.695 |
| Trust vet advice about disease control | Age: Over 60 | 0.04 | -0.16 - 0.25 | 0.693 |
| Farmers receive high quality vet advice | Intercept | 4.00 | 3.70 - 4.30 |  |
| Farmers receive high quality vet advice | Country: England | -0.10 | -0.35 - 0.16 | 0.451 |
| Farmers receive high quality vet advice | Country: Scotland | -0.01 | -0.31 - 0.29 | 0.957 |
| Farmers receive high quality vet advice | Herd type: Dairy | -0.04 | -0.18 - 0.10 | 0.572 |
| Farmers receive high quality vet advice | Age: 40-49 | -0.01 | -0.24 - 0.22 | 0.943 |
| Farmers receive high quality vet advice | Age: 50-59 | -0.02 | -0.23 - 0.18 | 0.838 |
| Farmers receive high quality vet advice | Age: Over 60 | 0.14 | -0.06 - 0.34 | 0.162 |
| Trust National Farmers Union | Intercept | 3.69 | 3.28 - 4.10 |  |
| Trust National Farmers Union | Country: England | -0.30 | -0.65 - 0.05 | 0.090 |
| Trust National Farmers Union | Country: Scotland | -0.45 | -0.86 - -0.04 | 0.032 |
| Trust National Farmers Union | Herd type: Dairy | 0.08 | -0.12 - 0.27 | 0.426 |
| Trust National Farmers Union | Age: 40-49 | 0.10 | -0.22 - 0.42 | 0.534 |
| Trust National Farmers Union | Age: 50-59 | 0.12 | -0.16 - 0.39 | 0.411 |
| Trust National Farmers Union | Age: Over 60 | 0.17 | -0.10 - 0.44 | 0.213 |
| Feels respected by National Farmers Union | Intercept | 3.71 | 3.31 - 4.11 |  |
| Feels respected by National Farmers Union | Country: England | -0.39 | -0.73 - -0.06 | 0.022 |
| Feels respected by National Farmers Union | Country: Scotland | -0.46 | -0.86 - -0.06 | 0.023 |
| Feels respected by National Farmers Union | Herd type: Dairy | 0.06 | -0.13 - 0.25 | 0.517 |
| Feels respected by National Farmers Union | Age: 40-49 | 0.12 | -0.18 - 0.43 | 0.426 |
| Feels respected by National Farmers Union | Age: 50-59 | -0.02 | -0.29 - 0.24 | 0.859 |
| Feels respected by National Farmers Union | Age: Over 60 | 0.09 | -0.18 - 0.35 | 0.517 |
| Trust governmental organisations | Intercept | 2.91 | 2.54 - 3.29 |  |
| Trust governmental organisations | Country: England | -0.12 | -0.43 - 0.20 | 0.462 |
| Trust governmental organisations | Country: Scotland | 0.19 | -0.18 - 0.57 | 0.305 |
| Trust governmental organisations | Herd type: Dairy | 0.08 | -0.09 - 0.26 | 0.347 |
| Trust governmental organisations | Age: 40-49 | -0.02 | -0.31 - 0.26 | 0.873 |
| Trust governmental organisations | Age: 50-59 | -0.25 | -0.50 - 0.00 | 0.052 |
| Trust governmental organisations | Age: Over 60 | -0.17 | -0.42 - 0.08 | 0.174 |
| Feels respected by Government | Intercept | 2.61 | 2.24 - 2.98 |  |
| Feels respected by Government | Country: England | -0.26 | -0.57 - 0.05 | 0.102 |
| Feels respected by Government | Country: Scotland | -0.14 | -0.51 - 0.23 | 0.454 |
| Feels respected by Government | Herd type: Dairy | 0.07 | -0.11 - 0.25 | 0.435 |
| Feels respected by Government | Age: 40-49 | -0.14 | -0.42 - 0.15 | 0.352 |
| Feels respected by Government | Age: 50-59 | -0.04 | -0.29 - 0.21 | 0.756 |
| Feels respected by Government | Age: Over 60 | -0.12 | -0.37 - 0.12 | 0.318 |
| Careful before trust Government | Intercept | 3.84 | 3.48 - 4.19 |  |
| Careful before trust Government | Country: England | -0.01 | -0.31 - 0.29 | 0.948 |
| Careful before trust Government | Country: Scotland | -0.26 | -0.62 - 0.09 | 0.148 |
| Careful before trust Government | Herd type: Dairy | 0.00 | -0.17 - 0.17 | 0.986 |
| Careful before trust Government | Age: 40-49 | 0.00 | -0.28 - 0.27 | 0.995 |
| Careful before trust Government | Age: 50-59 | -0.07 | -0.31 - 0.17 | 0.554 |
| Careful before trust Government | Age: Over 60 | -0.04 | -0.28 - 0.19 | 0.737 |
| Trust governmental judgements about disease control | Intercept | 2.34 | 1.93 - 2.75 |  |
| Trust governmental judgements about disease control | Country: England | -0.12 | -0.47 - 0.23 | 0.500 |
| Trust governmental judgements about disease control | Country: Scotland | 0.39 | -0.02 - 0.80 | 0.063 |
| Trust governmental judgements about disease control | Herd type: Dairy | 0.08 | -0.11 - 0.28 | 0.408 |
| Trust governmental judgements about disease control | Age: 40-49 | 0.03 | -0.29 - 0.34 | 0.859 |
| Trust governmental judgements about disease control | Age: 50-59 | 0.07 | -0.20 - 0.35 | 0.595 |
| Trust governmental judgements about disease control | Age: Over 60 | 0.13 | -0.14 - 0.40 | 0.355 |
| Psychological proximity to neighbouring farmers | Intercept | 3.60 | 2.91 - 4.28 |  |
| Psychological proximity to neighbouring farmers | Country: England | -0.09 | -0.67 - 0.49 | 0.753 |
| Psychological proximity to neighbouring farmers | Country: Scotland | 0.07 | -0.61 - 0.75 | 0.843 |
| Psychological proximity to neighbouring farmers | Herd type: Dairy | 0.08 | -0.24 - 0.41 | 0.618 |
| Psychological proximity to neighbouring farmers | Age: 40-49 | 0.36 | -0.17 - 0.89 | 0.181 |
| Psychological proximity to neighbouring farmers | Age: 50-59 | 0.35 | -0.11 - 0.81 | 0.133 |
| Psychological proximity to neighbouring farmers | Age: Over 60 | 0.48 | 0.03 - 0.93 | 0.038 |
| Psychological proximity to beef farmers | Intercept | 3.73 | 3.13 - 4.32 |  |
| Psychological proximity to beef farmers | Country: England | -0.34 | -0.84 - 0.16 | 0.186 |
| Psychological proximity to beef farmers | Country: Scotland | 0.11 | -0.48 - 0.71 | 0.712 |
| Psychological proximity to beef farmers | Herd type: Dairy | -0.59 | -0.87 - -0.30 | < 0.001 |
| Psychological proximity to beef farmers | Age: 40-49 | 0.23 | -0.23 - 0.69 | 0.321 |
| Psychological proximity to beef farmers | Age: 50-59 | 0.19 | -0.21 - 0.59 | 0.356 |
| Psychological proximity to beef farmers | Age: Over 60 | 0.14 | -0.26 - 0.54 | 0.489 |
| Psychological proximity to dairy farmers | Intercept | 3.08 | 2.46 - 3.71 |  |
| Psychological proximity to dairy farmers | Country: England | -0.12 | -0.65 - 0.41 | 0.654 |
| Psychological proximity to dairy farmers | Country: Scotland | -0.46 | -1.08 - 0.17 | 0.152 |
| Psychological proximity to dairy farmers | Herd type: Dairy | 0.91 | 0.61 - 1.21 | < 0.001 |
| Psychological proximity to dairy farmers | Age: 40-49 | -0.08 | -0.56 - 0.40 | 0.741 |
| Psychological proximity to dairy farmers | Age: 50-59 | 0.10 | -0.32 - 0.52 | 0.638 |
| Psychological proximity to dairy farmers | Age: Over 60 | 0.19 | -0.23 - 0.60 | 0.376 |
| Psychological proximity to farming community | Intercept | 3.77 | 3.18 - 4.35 |  |
| Psychological proximity to farming community | Country: England | -0.47 | -0.97 - 0.02 | 0.063 |
| Psychological proximity to farming community | Country: Scotland | -0.26 | -0.84 - 0.33 | 0.386 |
| Psychological proximity to farming community | Herd type: Dairy | -0.08 | -0.36 - 0.20 | 0.564 |
| Psychological proximity to farming community | Age: 40-49 | 0.08 | -0.36 - 0.53 | 0.714 |
| Psychological proximity to farming community | Age: 50-59 | 0.02 | -0.37 - 0.41 | 0.923 |
| Psychological proximity to farming community | Age: Over 60 | -0.06 | -0.45 - 0.32 | 0.755 |
| Psychological proximity to vet | Intercept | 4.87 | 4.16 - 5.58 |  |
| Psychological proximity to vet | Country: England | -0.16 | -0.76 - 0.45 | 0.612 |
| Psychological proximity to vet | Country: Scotland | 0.23 | -0.48 - 0.94 | 0.526 |
| Psychological proximity to vet | Herd type: Dairy | 0.46 | 0.12 - 0.80 | 0.008 |
| Psychological proximity to vet | Age: 40-49 | -0.23 | -0.78 - 0.32 | 0.405 |
| Psychological proximity to vet | Age: 50-59 | -0.22 | -0.70 - 0.27 | 0.381 |
| Psychological proximity to vet | Age: Over 60 | 0.01 | -0.47 - 0.48 | 0.974 |
| Psychological proximity to vet community | Intercept | 3.64 | 2.96 - 4.32 |  |
| Psychological proximity to vet community | Country: England | -0.68 | -1.26 - -0.10 | 0.023 |
| Psychological proximity to vet community | Country: Scotland | -0.52 | -1.21 - 0.16 | 0.134 |
| Psychological proximity to vet community | Herd type: Dairy | 0.16 | -0.16 - 0.48 | 0.320 |
| Psychological proximity to vet community | Age: 40-49 | 0.09 | -0.43 - 0.61 | 0.727 |
| Psychological proximity to vet community | Age: 50-59 | 0.38 | -0.07 - 0.83 | 0.102 |
| Psychological proximity to vet community | Age: Over 60 | 0.67 | 0.22 - 1.12 | 0.004 |
| Psychological proximity to National Farmers Union | Intercept | 2.72 | 2.05 - 3.38 |  |
| Psychological proximity to National Farmers Union | Country: England | -0.29 | -0.85 - 0.27 | 0.313 |
| Psychological proximity to National Farmers Union | Country: Scotland | -0.63 | -1.29 - 0.03 | 0.063 |
| Psychological proximity to National Farmers Union | Herd type: Dairy | 0.16 | -0.16 - 0.47 | 0.329 |
| Psychological proximity to National Farmers Union | Age: 40-49 | 0.19 | -0.32 - 0.70 | 0.458 |
| Psychological proximity to National Farmers Union | Age: 50-59 | 0.57 | 0.12 - 1.01 | 0.013 |
| Psychological proximity to National Farmers Union | Age: Over 60 | 0.88 | 0.44 - 1.32 | < 0.001 |
| Psychological proximity to government | Intercept | 2.00 | 1.49 - 2.52 |  |
| Psychological proximity to government | Country: England | -0.36 | -0.79 - 0.07 | 0.105 |
| Psychological proximity to government | Country: Scotland | -0.01 | -0.53 - 0.50 | 0.959 |
| Psychological proximity to government | Herd type: Dairy | 0.07 | -0.18 - 0.31 | 0.594 |
| Psychological proximity to government | Age: 40-49 | 0.18 | -0.22 - 0.58 | 0.373 |
| Psychological proximity to government | Age: 50-59 | 0.24 | -0.10 - 0.59 | 0.170 |
| Psychological proximity to government | Age: Over 60 | 0.35 | 0.01 - 0.69 | 0.045 |
| Psychological proximity to cows | Intercept | 5.82 | 5.26 - 6.38 |  |
| Psychological proximity to cows | Country: England | 0.48 | 0.01 - 0.96 | 0.045 |
| Psychological proximity to cows | Country: Scotland | 0.30 | -0.26 - 0.85 | 0.299 |
| Psychological proximity to cows | Herd type: Dairy | 0.11 | -0.16 - 0.38 | 0.418 |
| Psychological proximity to cows | Age: 40-49 | -0.17 | -0.60 - 0.26 | 0.447 |
| Psychological proximity to cows | Age: 50-59 | -0.25 | -0.63 - 0.12 | 0.189 |
| Psychological proximity to cows | Age: Over 60 | -0.34 | -0.72 - 0.03 | 0.070 |
| Psychological capability | Intercept | 4.20 | 3.99 - 4.42 |  |
| Psychological capability | Country: England | 0.09 | -0.09 - 0.27 | 0.321 |
| Psychological capability | Country: Scotland | 0.18 | -0.04 - 0.39 | 0.106 |
| Psychological capability | Herd type: Dairy | 0.07 | -0.03 - 0.17 | 0.190 |
| Psychological capability | Age: 40-49 | -0.02 | -0.18 - 0.15 | 0.837 |
| Psychological capability | Age: 50-59 | -0.14 | -0.28 - 0.01 | 0.061 |
| Psychological capability | Age: Over 60 | -0.14 | -0.28 - 0.00 | 0.043 |
| Physical opportunity | Intercept | 3.72 | 3.39 - 4.05 |  |
| Physical opportunity | Country: England | 0.13 | -0.14 - 0.41 | 0.351 |
| Physical opportunity | Country: Scotland | 0.28 | -0.05 - 0.61 | 0.095 |
| Physical opportunity | Herd type: Dairy | 0.02 | -0.14 - 0.17 | 0.845 |
| Physical opportunity | Age: 40-49 | 0.07 | -0.18 - 0.32 | 0.594 |
| Physical opportunity | Age: 50-59 | 0.01 | -0.21 - 0.23 | 0.926 |
| Physical opportunity | Age: Over 60 | 0.14 | -0.08 - 0.35 | 0.213 |
| Social opportunity | Intercept | 3.27 | 3.07 - 3.46 |  |
| Social opportunity | Country: England | 0.04 | -0.12 - 0.20 | 0.609 |
| Social opportunity | Country: Scotland | 0.26 | 0.07 - 0.45 | 0.009 |
| Social opportunity | Herd type: Dairy | 0.10 | 0.01 - 0.19 | 0.036 |
| Social opportunity | Age: 40-49 | 0.07 | -0.08 - 0.22 | 0.373 |
| Social opportunity | Age: 50-59 | -0.03 | -0.16 - 0.10 | 0.614 |
| Social opportunity | Age: Over 60 | 0.05 | -0.08 - 0.17 | 0.450 |
| Automatic motivation | Intercept | 4.48 | 4.25 - 4.70 |  |
| Automatic motivation | Country: England | -0.07 | -0.26 - 0.12 | 0.474 |
| Automatic motivation | Country: Scotland | -0.09 | -0.31 - 0.14 | 0.452 |
| Automatic motivation | Herd type: Dairy | -0.08 | -0.19 - 0.02 | 0.127 |
| Automatic motivation | Age: 40-49 | -0.10 | -0.28 - 0.07 | 0.252 |
| Automatic motivation | Age: 50-59 | -0.19 | -0.34 - -0.04 | 0.015 |
| Automatic motivation | Age: Over 60 | -0.14 | -0.29 - 0.01 | 0.066 |
| Reflective motivation | Intercept | 4.45 | 4.23 - 4.67 |  |
| Reflective motivation | Country: England | 0.00 | -0.19 - 0.19 | 0.998 |
| Reflective motivation | Country: Scotland | 0.06 | -0.16 - 0.28 | 0.591 |
| Reflective motivation | Herd type: Dairy | 0.04 | -0.06 - 0.15 | 0.402 |
| Reflective motivation | Age: 40-49 | -0.08 | -0.25 - 0.09 | 0.366 |
| Reflective motivation | Age: 50-59 | -0.16 | -0.30 - -0.01 | 0.040 |
| Reflective motivation | Age: Over 60 | -0.09 | -0.23 - 0.06 | 0.240 |

Supplementary Table 3. Post-hoc analysis of the effect size for which a difference could be detected between two countries at 0.0015 significance level and 80% power.

| Factor | | Wales & Scotland | Wales & England | Scotland & England |
| --- | --- | --- | --- | --- |
| Altruism | Social value orientation | 11.15 | 9.55 | 6.63 |
| Trust | Trust beef farmers | 0.66 | 0.56 | 0.42 |
|  | Trust dairy farmers | 0.65 | 0.55 | 0.40 |
|  | Trust farmers met for first time | 0.64 | 0.54 | 0.40 |
|  | Careful before trust farmers | 0.74 | 0.62 | 0.46 |
|  | Trust neighbours to control disease | 0.83 | 0.70 | 0.52 |
|  | Trust farmers to control disease | 0.76 | 0.64 | 0.47 |
|  | Trust vets | 0.61 | 0.52 | 0.38 |
|  | Feels respected by vet | 0.63 | 0.53 | 0.39 |
|  | Feels respected by vets | 0.72 | 0.61 | 0.45 |
|  | Careful before trust vets | 0.86 | 0.72 | 0.54 |
|  | Vet would always tell truth | 0.61 | 0.51 | 0.38 |
|  | Trust vet advice about disease control | 0.63 | 0.53 | 0.39 |
|  | Farmers receive high quality vet advice | 0.62 | 0.52 | 0.39 |
|  | Trust National Farmers Union | 0.84 | 0.71 | 0.53 |
|  | Feels respected by National Farmers Union | 0.82 | 0.69 | 0.51 |
|  | Trust governmental organisations | 0.77 | 0.65 | 0.48 |
|  | Feels respected by Government | 0.76 | 0.64 | 0.47 |
|  | Careful before trust Government | 0.74 | 0.62 | 0.46 |
|  | Trust governmental judgements about disease control | 0.86 | 0.72 | 0.53 |
| Psychological proximity | Psychological proximity to neighbouring farmers | 1.40 | 1.17 | 0.87 |
|  | Psychological proximity to beef farmers | 1.25 | 1.05 | 0.78 |
|  | Psychological proximity to dairy farmers | 1.34 | 1.13 | 0.84 |
|  | Psychological proximity to farming community | 1.20 | 1.01 | 0.74 |
|  | Psychological proximity to vet | 1.46 | 1.23 | 0.91 |
|  | Psychological proximity to vet community | 1.42 | 1.20 | 0.87 |
|  | Psychological proximity to National Farmers Union | 1.39 | 1.17 | 0.87 |
|  | Psychological proximity to government | 1.06 | 0.89 | 0.67 |
|  | Psychological proximity to cows | 1.15 | 0.97 | 0.72 |
| COM-B | Psychological capability | 0.44 | 0.37 | 0.27 |
|  | Physical opportunity | 0.67 | 0.56 | 0.42 |
|  | Social opportunity | 0.40 | 0.33 | 0.25 |
|  | Automatic motivation | 0.46 | 0.39 | 0.29 |
|  | Reflective motivation | 0.45 | 0.38 | 0.28 |

Supplementary Table 4. Post-hoc analysis of the effect size for which a difference could be detected between two countries at 0.05 significance level and 80% power.

| Factor | Wales & Scotland | Wales & England | Scotland & England |
| --- | --- | --- | --- |
| Social value orientation | 7.65 | 6.63 | 4.61 |
| Trust beef farmers | 0.46 | 0.39 | 0.29 |
| Trust dairy farmers | 0.45 | 0.38 | 0.28 |
| Trust farmers met for first time | 0.44 | 0.38 | 0.28 |
| Careful before trust farmers | 0.51 | 0.43 | 0.32 |
| Trust neighbours to control disease | 0.57 | 0.49 | 0.36 |
| Trust farmers to control disease | 0.52 | 0.44 | 0.33 |
| Trust vets | 0.42 | 0.36 | 0.27 |
| Feels respected by vet | 0.43 | 0.37 | 0.27 |
| Feels respected by vets | 0.49 | 0.42 | 0.31 |
| Careful before trust vets | 0.59 | 0.50 | 0.37 |
| Vet would always tell truth | 0.42 | 0.36 | 0.26 |
| Trust vet advice about disease control | 0.43 | 0.37 | 0.27 |
| Farmers receive high quality vet advice | 0.43 | 0.36 | 0.27 |
| Trust National Farmers Union | 0.58 | 0.49 | 0.37 |
| Feels respected by National Farmers Union | 0.56 | 0.48 | 0.36 |
| Trust governmental organisations | 0.53 | 0.45 | 0.33 |
| Feels respected by Government | 0.52 | 0.44 | 0.33 |
| Careful before trust Government | 0.51 | 0.43 | 0.32 |
| Trust governmental judgements about disease control | 0.59 | 0.50 | 0.37 |
| Psychological proximity to neighbouring farmers | 0.96 | 0.82 | 0.61 |
| Psychological proximity to beef farmers | 0.86 | 0.73 | 0.54 |
| Psychological proximity to dairy farmers | 0.92 | 0.78 | 0.58 |
| Psychological proximity to farming community | 0.82 | 0.70 | 0.51 |
| Psychological proximity to vet | 1.00 | 0.85 | 0.63 |
| Psychological proximity to vet community | 0.98 | 0.84 | 0.61 |
| Psychological proximity to National Farmers Union | 0.96 | 0.81 | 0.61 |
| Psychological proximity to government | 0.73 | 0.62 | 0.46 |
| Psychological proximity to cows | 0.79 | 0.67 | 0.50 |
| Psychological capability | 0.30 | 0.26 | 0.19 |
| Physical opportunity | 0.46 | 0.39 | 0.29 |
| Social opportunity | 0.27 | 0.23 | 0.17 |
| Automatic motivation | 0.32 | 0.27 | 0.20 |
| Reflective motivation | 0.31 | 0.26 | 0.20 |

Supplementary Figure 1. The total within-cluster sum of squares for 1 to 10 clusters of the psychosocial and COM-B scores of 389 cattle farmers using k-means clustering of the factors.


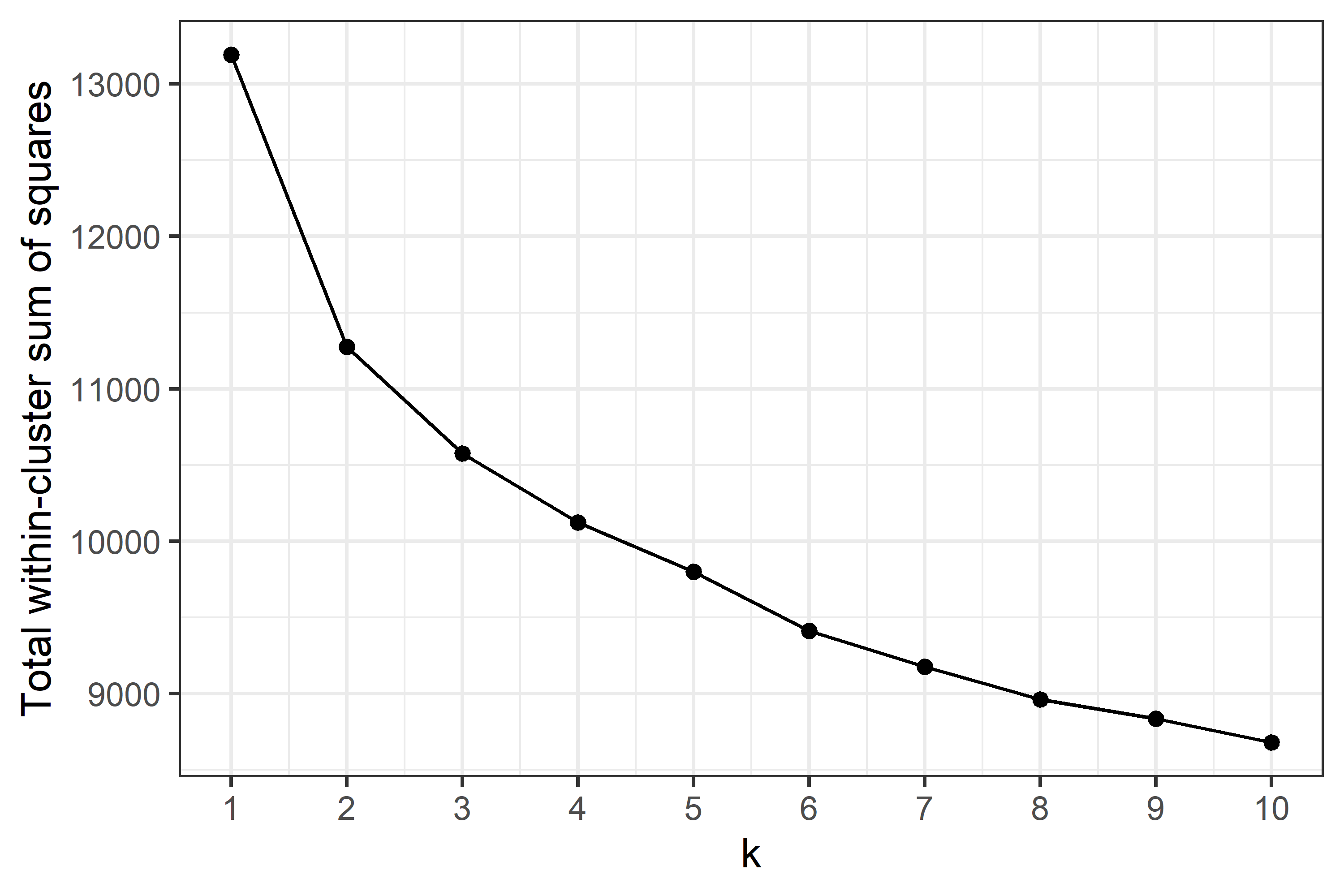


*Supplementary Information 1. Questions used in the survey to assess each factor in the COM-B behaviour change framework.*

**Psychological capability**

I know how to control infectious disease in my cattle

I know why it is important to control infectious disease in my cattle

I understand most advice I receive about infectious disease in cattle

**Physical Opportunity**

I do not have the time to control infectious disease in my cattle

Controlling infectious disease costs too much money

**Social Opportunity**

Most farmers I know are controlling infectious disease in their cattle

I find it difficult to raise the subject of infectious disease in my cattle with other farmers

My vet helps me control infectious diseases in my cattle

Government policy helps me control infectious disease in my cattle

Other farmers help me achieve control of infectious disease in my cattle

I find it difficult to raise the subject of infectious disease in my cattle with vets

I find it difficult to raise the subject of infectious disease in my cattle with governmental organisations

**Automatic Motivation**

I worry about getting infectious diseases in my cattle

I feel good about myself when I control infectious disease in my cattle

I want to control infectious diseases for the sake of my herd

Controlling infectious diseases in my cattle is part of my routine

**Reflective Motivation**

I have infectious disease goals that I want to achieve for my herd

I want to control infectious disease in my cattle

There are many benefits to controlling infectious disease in cattle

It is not my responsibility to control infectious disease in my cattle

I have a plan for controlling infectious disease in my cattle
