# Supplementary material for: Between‐country differences in the psychosocial profiles of British cattle farmers
Source: Vet Rec. 2025 Oct 12;198(4):e159–65. doi: 10.1002/vetr.5672 (PMC12904086; doi:10.1002/vetr.5672)
Supplement: Supplementary file 2 — Supporting Information [file VETR-198--s002.pdf]

```
### Psychological profiles by region ###
# R version 4.2.2
# Date 15/08/23

#### Load packages ####
library(dplyr)
library(ggplot2)
library(data.table)
library(lme4) # multilevel model
library(tidyr)
library(tidyverse)
library(ggraph) # For network plot
library(igraph) # For network plot
library(RColorBrewer) # For colour palettes
library(pwr) # For power analysis

#### Load data ####
Data <- read.csv("./Clean_Data_with_factors_and_LCA_11_03_21.csv", na.strings=c("","NA"))
Data <- subset(Data, Country!="NI")

#### Make data correct format for the analysis ####

# Correct country data
Data$Country[Data$Q4=="Berwickshire"] <- "Sco"

## Select columns of interest
Keep_cols <- c("Country", "Q3", "Q5", "Q6", "Q11", "Q13", "Q14_1", "SVODegrees", "Q30_1", "Q30_2", "Q30_3", "Q30_4", "Q30_5", "Q30_6", "Q30_7", "Q30_8", "Q30_9",
"Q30_10", "Q30_11", "Q30_12", "Q30_13", "Q30_14", "Q30_15", "Q30_17", "Q30_18", "Q30_19", "Q30_21", "Q31_1", "Q31_2", "Q31_3", "Q31_4", "Q31_5", "Q31_6",
"Q31_7", "Q31_8", "Q31_9", "Psychological_capability", "Physical_opportunity", "Social_opportunity", "Automatic_motivation", "Reflective_motivation")
Data <- Data[,Keep_cols]
colnames(Data) <- c("Country", "Herd_type", "Age", "Routine_vet_visits", "N_dairy", "N_beef", "Suckler_cows", "SVODegrees", "Trust_dairy_farmers",
"Feels_respected_by_Gov", "Trust_gov_judgements_about_disease_control", "Careful_before_trust_farmers", "Trust_farmers_to_control_disease",
"Feel_respected_by_vet", "Trust_NFU", "Careful_before_trust_vets", "Trust_farmers_met_for_first_time", "Farmers_receive_high_quality_vet_advice",
"Careful_before_trust_Gov", "Trust_neighbours_to_control_disease", "Trust_vets", "Feel_respected_by_NFU", "Trust_beef_farmers", "Feel_respected_by_vets",
"Vet_would_always_tell_truth", "Trust_vet_advice_about_disease_control", "Trust_gov_orgs", "Psychological_proximity_to_vet",
"Psychological_proximity_to_vet_community", "Psychological_proximity_to_neighbouring_farmers", "Psychological_proximity_to_farming_community",
"Psychological_proximity_to_government", "Psychological_proximity_to_NFU", "Psychological_proximity_to_cows", "Psychological_proximity_to_dairy_farmers",
"Psychological_proximity_to_beef_farmers", "Psychological_capability", "Physical_opportunity", "Social_opportunity", "Automatic_motivation", "Reflective_motivation")
```

```
# Make "Herd_type" a factor
Data$Herd_type <- as.factor(Data$Herd_type)
levels(Data$Herd_type) <- list("Beef only" = "Beef", "Dairy" = c("Beef and Dairy", "Dairy"))
# Make "Suckler_cows" a factor
Data$Suckler_cows <- as.factor(Data$Suckler_cows)
# Make "Age" a factor
Data$Age <- as.factor(Data$Age)
levels(Data$Age) <- list("Under 30" = "Under 30", "30-39" = "30-39", "40-49" = "40-49", "50-59" = "50-59", "60-69" = "60-69", "Over 70" = "Over 70")
# Make "Routine_vet_visits" a factor
Data$Routine_vet_visits <- as.factor(Data$Routine_vet_visits)
# Make NA herd size 0
Data$N_dairy[is.na(Data$N_dairy)] <- 0
Data$N_beef[is.na(Data$N_beef)] <- 0

# Combine age categories to ensure at least 5 in each
levels(Data$Age) <- list("Under 40" = c("Under 30", "30-39"), "40-49" = "40-49", "50-59" = "50-59", "Over 60" = c("60-69", "Over 70"))

# Make country a factor
Data$Country <- as.factor(Data$Country)

## Make trust questions numeric
# Function
AgreementScale <- function(x){
  levels(x) <- list("1"="Strongly disagree", "2"="Disagree", "3"="Neither agree nor disagree", "4"="Agree", "5"="Strongly agree")
  x <- x %>% as.character() %>% as.numeric
  return(x)
}
Trust_Qs <- c("Trust_dairy_farmers", "Feels_respected_by_Gov", "Trust_gov_judgements_about_disease_control", "Careful_before_trust_farmers",
"Trust_farmers_to_control_disease", "Feel_respected_by_vet", "Trust_NFU", "Careful_before_trust_vets", "Trust_farmers_met_for_first_time",
"Farmers_receive_high_quality_vet_advice", "Careful_before_trust_Gov", "Trust_neighbours_to_control_disease", "Trust_vets", "Feel_respected_by_NFU",
"Trust_beef_farmers", "Feel_respected_by_vets", "Vet_would_always_tell_truth", "Trust_vet_advice_about_disease_control", "Trust_gov_orgs")

Data[, Trust_Qs] <- lapply(Data[, Trust_Qs], as.factor)
Data[, Trust_Qs] <- lapply(Data[, Trust_Qs], AgreementScale)

## Save lists of variable categories
```

```
Trust_variables <- c("Trust_dairy_farmers", "Feels_respected_by_Gov", "Trust_gov_judgements_about_disease_control", "Careful_before_trust_farmers",
"Trust_farmers_to_control_disease", "Feel_respected_by_vet", "Trust_NFU", "Careful_before_trust_vets", "Trust_farmers_met_for_first_time",
"Farmers_receive_high_quality_vet_advice", "Careful_before_trust_Gov", "Trust_neighbours_to_control_disease", "Trust_vets", "Feel_respected_by_NFU",
"Trust_beef_farmers", "Feel_respected_by_vets", "Vet_would_always_tell_truth", "Trust_vet_advice_about_disease_control", "Trust_gov_orgs")
Psychological_proximity_variables <- c("Psychological_proximity_to_vet", "Psychological_proximity_to_vet_community",
"Psychological_proximity_to_neighbouring_farmers", "Psychological_proximity_to_farming_community", "Psychological_proximity_to_government",
"Psychological_proximity_to_NFU", "Psychological_proximity_to_cows", "Psychological_proximity_to_dairy_farmers", "Psychological_proximity_to_beef_farmers")
Psychological_proximity_variables_scaled <- c("Psychological_proximity_to_vet_scaled", "Psychological_proximity_to_vet_community_scaled",
"Psychological_proximity_to_neighbouring_farmers_scaled", "Psychological_proximity_to_farming_community_scaled",
"Psychological_proximity_to_government_scaled", "Psychological_proximity_to_NFU_scaled", "Psychological_proximity_to_cows_scaled",
"Psychological_proximity_to_dairy_farmers_scaled", "Psychological_proximity_to_beef_farmers_scaled")
COMB_variables <- c("Psychological_capability", "Physical_opportunity", "Social_opportunity", "Automatic_motivation", "Reflective_motivation")

### Put variables on same scale
# SVO
Data$SVO_scaled <- ((Data$SVODegrees + 16.26) / (77.65/4)) + 1
# Psychological proximity
PP_scaled <- function(x){
  x <- ((x - 1) / 1.5) + 1
  return(x)
}
# Normalise
Scaled_PP_data <- lapply(Data[,Psychological_proximity_variables], PP_scaled) %>% as.data.frame()
# Identify rescaled data in the column name and attach data to dataframe
colnames(Scaled_PP_data) <- paste(colnames(Scaled_PP_data), "scaled", sep = "_")
Data <- cbind(Data, Scaled_PP_data)

## Create list of dependent variables for models
Dependent_variables <- c("SVODegrees",
                        "Trust_beef_farmers", "Trust_dairy_farmers", "Trust_farmers_met_for_first_time", "Careful_before_trust_farmers", "Trust_neighbours_to_control_disease",
"Trust_farmers_to_control_disease",
                        "Trust_vets", "Feel_respected_by_vet", "Feel_respected_by_vets", "Careful_before_trust_vets", "Vet_would_always_tell_truth",
"Trust_vet_advice_about_disease_control", "Farmers_receive_high_quality_vet_advice",
                        "Trust_NFU", "Feel_respected_by_NFU",
                        "Trust_gov_orgs", "Feels_respected_by_Gov", "Careful_before_trust_Gov", "Trust_gov_judgements_about_disease_control",
                        "Psychological_proximity_to_neighbouring_farmers", "Psychological_proximity_to_beef_farmers", "Psychological_proximity_to_dairy_farmers",
"Psychological_proximity_to_farming_community",
                        "Psychological_proximity_to_vet", "Psychological_proximity_to_vet_community",
```

```
                    "Psychological_proximity_to_NFU",
                    "Psychological_proximity_to_government",
                    "Psychological_proximity_to_cows",
                    "Psychological_capability", "Physical_opportunity", "Social_opportunity", "Automatic_motivation", "Reflective_motivation")

#### Calculate correlations ####
Correlations <- as.data.frame(cor(Data[,c("SVODegrees", Trust_variables, Psychological_proximity_variables, COMB_variables)], use="complete.obs"))
write.csv(Correlations,"Correlations.csv", na="", row.names=TRUE)

#### Country analysis ####

## Run & save models with Wales as reference category
levels(Data$Country) <- list("Wal" = "Wal", "Eng" = "Eng", "Sco" = "Sco")

Country_model_Wal_ref_list <- list()
for(i in 1:length(Dependent_variables)){
  j <- Dependent_variables[i]
  model <- glm(formula = paste0(j, "~ Country"), data = Data)
  Coefs_P_model <- summary(model)$coefficients[,c(1,4)] %>%
    as.data.frame()
  colnames(Coefs_P_model) <- c("Coefficient", "P")
  Coefs_P_model$Coefficient <- formatC(Coefs_P_model$Coefficient, digits = 2, format = "f")
  Coefs_P_model$P <- formatC(Coefs_P_model$P, digits = 3, format = "f")
  CIs_model <- confint(model) %>%
    round(digits = 2) %>%
    formatC(digits = 2, format = "f") %>%
    as.data.frame()
  colnames(CIs_model) <- c("low_CI", "high_CI")
  CIs_model$CI <- paste(CIs_model$low_CI, CIs_model$high_CI, sep = " - ")
  model_results <- cbind(Coefs_P_model, CIs_model$CI)
  colnames(model_results) <- c(colnames(model_results[1:2]), "CI")
  model_results$Model <- j
  model_results$Variable <- row.names(model_results)
  model_results <- model_results %>%
    select(Model, Variable, Coefficient, CI, P)
  Country_model_Wal_ref_list[[i]] <- model_results
}
rm(i, j, model, Coefs_P_model, CIs_model, model_results)
```

```
Country_model_Wal_ref_list <- do.call(rbind, Country_model_Wal_ref_list)
write.csv(Country_model_Wal_ref_list, "Country_models_with_Wales_reference.csv", row.names = FALSE)

## Run and save models with Scotland as reference category
levels(Data$Country) <- list("Sco" = "Sco", "Eng" = "Eng", "Wal" = "Wal")

Country_model_Sco_ref_list <- list()
for(i in 1:length(Dependent_variables)){
  j <- Dependent_variables[i]
  model <- glm(formula = paste0(j, "~ Country"), data = Data)
  Coefs_P_model <- summary(model)$coefficients[,c(1,4)] %>%
    as.data.frame()
  colnames(Coefs_P_model) <- c("Coefficient", "P")
  Coefs_P_model$Coefficient <- formatC(Coefs_P_model$Coefficient, digits = 2, format = "f")
  Coefs_P_model$P <- formatC(Coefs_P_model$P, digits = 3, format = "f")
  CIs_model <- confint(model) %>%
    round(digits = 2) %>%
    formatC(digits = 2, format = "f") %>%
    as.data.frame()
  colnames(CIs_model) <- c("low_CI", "high_CI")
  CIs_model$CI <- paste(CIs_model$low_CI, CIs_model$high_CI, sep = " - ")
  model_results <- cbind(Coefs_P_model, CIs_model$CI)
  colnames(model_results) <- c(colnames(model_results[1:2]), "CI")
  model_results$Model <- j
  model_results$Variable <- row.names(model_results)
  model_results <- model_results %>%
    select(Model, Variable, Coefficient, CI, P)
  Country_model_Sco_ref_list[[i]] <- model_results
}
rm(i, j, model, Coefs_P_model, CIs_model, model_results)

Country_model_Sco_ref_list <- do.call(rbind, Country_model_Sco_ref_list)
write.csv(Country_model_Sco_ref_list, "Country_models_with_Scotland_reference.csv", row.names = FALSE)

#### Country analysis with possible confounders (age & herd type) ####

## Run & save models with Wales as reference category
```

```
levels(Data$Country) <- list("Wal" = "Wal", "Eng" = "Eng", "Sco" = "Sco")

Country_model_Wal_ref_inc_conf_list <- list()
for(i in 1:length(Dependent_variables)){
  j <- Dependent_variables[i]
  model <- glm(formula = paste0(j, "~ Country + Herd_type + Age"), data = Data)
  Coefs_P_model <- summary(model)$coefficients[,c(1,4)] %>%
    as.data.frame()
  colnames(Coefs_P_model) <- c("Coefficient", "P")
  Coefs_P_model$Coefficient <- formatC(Coefs_P_model$Coefficient, digits = 2, format = "f")
  Coefs_P_model$P <- formatC(Coefs_P_model$P, digits = 3, format = "f")
  CIs_model <- confint(model) %>%
    round(digits = 2) %>%
    formatC(digits = 2, format = "f") %>%
    as.data.frame()
  colnames(CIs_model) <- c("low_CI", "high_CI")
  CIs_model$CI <- paste(CIs_model$low_CI, CIs_model$high_CI, sep = " - ")
  model_results <- cbind(Coefs_P_model, CIs_model$CI)
  colnames(model_results) <- c(colnames(model_results[1:2]), "CI")
  model_results$Model <- j
  model_results$Variable <- row.names(model_results)
  model_results <- model_results %>%
    select(Model, Variable, Coefficient, CI, P)
  Country_model_Wal_ref_inc_conf_list[[i]] <- model_results
}
rm(i, j, model, Coefs_P_model, CIs_model, model_results)

Country_model_Wal_ref_inc_conf_list <- do.call(rbind, Country_model_Wal_ref_inc_conf_list)
write.csv(Country_model_Wal_ref_inc_conf_list, "Country_models_with_Wales_reference_and_confounders.csv", row.names = FALSE)

## Run & save models with Scotland as reference category
levels(Data$Country) <- list("Sco" = "Sco", "Eng" = "Eng", "Wal" = "Wal")

Country_model_Sco_ref_inc_conf_list <- list()
for(i in 1:length(Dependent_variables)){
  j <- Dependent_variables[i]
  model <- glm(formula = paste0(j, "~ Country + Herd_type + Age"), data = Data)
  Coefs_P_model <- summary(model)$coefficients[,c(1,4)] %>%
```

```
    as.data.frame()
  colnames(Coefs_P_model) <- c("Coefficient", "P")
  Coefs_P_model$Coefficient <- formatC(Coefs_P_model$Coefficient, digits = 2, format = "f")
  Coefs_P_model$P <- formatC(Coefs_P_model$P, digits = 3, format = "f")
  CIs_model <- confint(model) %>%
    round(digits = 2) %>%
    formatC(digits = 2, format = "f") %>%
    as.data.frame()
  colnames(CIs_model) <- c("low_CI", "high_CI")
  CIs_model$CI <- paste(CIs_model$low_CI, CIs_model$high_CI, sep = " - ")
  model_results <- cbind(Coefs_P_model, CIs_model$CI)
  colnames(model_results) <- c(colnames(model_results[1:2]), "CI")
  model_results$Model <- j
  model_results$Variable <- row.names(model_results)
  model_results <- model_results %>%
    select(Model, Variable, Coefficient, CI, P)
  Country_model_Sco_ref_inc_conf_list[[i]] <- model_results
}
rm(i, j, model, Coefs_P_model, CIs_model, model_results)

Country_model_Sco_ref_inc_conf_list <- do.call(rbind, Country_model_Sco_ref_inc_conf_list)
write.csv(Country_model_Sco_ref_inc_conf_list, "Country_models_with_Scotland_reference_and_confounders.csv", row.names = FALSE)

#### Country differences plot ####
## Only differences at p < 0.05

Sig_variables <- c("SVO_scaled", "Trust_gov_judgements_about_disease_control", "Trust_NFU", "Careful_before_trust_Gov", "Trust_vets", "Feel_respected_by_NFU",
"Trust_gov_orgs", "Psychological_proximity_to_vet_community_scaled", "Psychological_proximity_to_cows_scaled", "Psychological_proximity_to_beef_farmers_scaled",
"Social_opportunity")

# On same plot
Plot_data <- NULL
for(i in colnames(Data[,Sig_variables])){
  for(j in levels(Data$Country)){
    x <- Data[,c("Country", i)]
    x <- x[complete.cases(x),]
    x <- subset(x, Country==j)
    n <- nrow(x)
```

```
    mean <- mean(x[,i])
    sd <- sd(x[,i])
    margin <- qt(0.975, df = n - 1)* sd / sqrt(n)
    LCI <- mean - margin
    UCI <- mean + margin
    Plot_data <- rbind(Plot_data, c(i, j, mean, LCI, UCI))
    colnames(Plot_data) <- c("Variable", "Country", "Mean", "LCI", "UCI")
  }
}
Plot_data <- as.data.frame(Plot_data)
Plot_data[,c("Variable", "Country")] <- lapply(Plot_data[,c("Variable", "Country")], as.factor)
Plot_data[,c("Mean", "LCI", "UCI")] <- lapply(Plot_data[,c("Mean", "LCI", "UCI")], as.numeric)
Plot_data <- Plot_data[complete.cases(Plot_data),]
# Reorder and rephrase variables
levels(Plot_data$Variable) <- list("Social opportunity" = "Social_opportunity",
                                   "Psychological proximity to government" = "Psychological_proximity_to_government_scaled",
                                   "Psychological proximity to National Farmers Union" =  "Psychological_proximity_to_NFU_scaled",
                                   "Psychological proximity to veterinarians" = "Psychological_proximity_to_vet_community_scaled",
                                   "Psychological proximity to dairy farmers" = "Psychological_proximity_to_dairy_farmers_scaled",
                                   "Psychological proximity to beef farmers" = "Psychological_proximity_to_beef_farmers_scaled",
                                   "Psychological proximity to cows" = "Psychological_proximity_to_cows_scaled",
                                   "Trust gov. disease control decisions" = "Trust_gov_judgements_about_disease_control",
                                   "Careful before trusting government" = "Careful_before_trust_Gov",
                                   "Trust governmental organisations" = "Trust_gov_orgs",
                                   "Feel respected by National Farmers Union" = "Feel_respected_by_NFU",
                                   "Trust National Farmers Union" = "Trust_NFU",
                                   "Careful before trusting veterinarians" = "Careful_before_trust_vets",
                                   "Trust veterinarians" = "Trust_vets",
                                   "Social value orientation" = "SVO_scaled")
levels(Plot_data$Country) <- list("England" = "Eng",
                                  "Scotland" = "Sco",
                                  "Wales" =  "Wal")
## Create plot

# Format some terms to be bold (p<0.0015)
breaks <- levels(Plot_data$Variable)
labels <- as.expression(breaks)
labels[[1]] <- bquote(bold(.(labels[[1]])))
```

```
labels[[8]] <- bquote(bold(.(labels[[8]])))

png(filename="Sig_variables_by_country.png", res=600, width=5500, height=3000)
ggplot(data = Plot_data, aes(y = Mean, x = Variable, colour = Country)) +
  geom_point(aes(shape = Country), position = position_dodge2(width = 0.9, reverse = TRUE)) +
  geom_errorbar(aes(ymax = UCI, ymin = LCI), position = position_dodge2(width = 0.3, reverse = TRUE)) +
  ylim(1, 5) +
  labs(y = "Score") +
  scale_colour_manual(values = c("#FF0000", "#4169E1", "#00CD00")) +
  scale_x_discrete(label = labels, breaks = breaks) +
  theme_bw() +
  coord_flip()
dev.off()

#### Clustering ####

## Normalise terms for clustering

# Select variables of interest and remove NAs
Cluster_variables <- c("SVODegrees", "Trust_dairy_farmers", "Feels_respected_by_Gov", "Trust_gov_judgements_about_disease_control",
"Careful_before_trust_farmers", "Trust_farmers_to_control_disease", "Feel_respected_by_vet", "Trust_NFU", "Careful_before_trust_vets",
"Trust_farmers_met_for_first_time", "Farmers_receive_high_quality_vet_advice", "Careful_before_trust_Gov", "Trust_neighbours_to_control_disease", "Trust_vets",
"Feel_respected_by_NFU", "Trust_beef_farmers", "Feel_respected_by_vets", "Vet_would_always_tell_truth", "Trust_vet_advice_about_disease_control",
"Trust_gov_orgs", "Psychological_proximity_to_vet", "Psychological_proximity_to_vet_community", "Psychological_proximity_to_neighbouring_farmers",
"Psychological_proximity_to_farming_community", "Psychological_proximity_to_government", "Psychological_proximity_to_NFU", "Psychological_proximity_to_cows",
"Psychological_proximity_to_dairy_farmers", "Psychological_proximity_to_beef_farmers", "Psychological_capability", "Physical_opportunity", "Social_opportunity",
"Automatic_motivation", "Reflective_motivation")
Data <- Data[complete.cases(Data[, Cluster_variables]), ]

# Normalise
Norm_data <- scale(Data[, Cluster_variables]) %>% as.data.frame()
# Identify normalised data in the column name and attach data to dataframe
colnames(Norm_data) <- paste(colnames(Norm_data), "norm", sep = "_")
Data <- cbind(Data, Norm_data)
# Update selected variables to normalised versions
Cluster_variables <- paste(Cluster_variables, "norm", sep = "_")

## K-means clustering
```

```
## Use map_dbl to run many models with varying value of k (centers)
set.seed(1612)
tot_withinss <- map_dbl(1:10,  function(k){
  model <- kmeans(x = Data[, Cluster_variables], centers = k)
  model$tot.withinss
})
## Generate a data frame containing both k and tot_withinss
elbow_df <- data.frame(
  k = 1:10,
  tot_withinss = tot_withinss
)
## Plot the elbow plot
png(filename="Psychosocial_clustering_elbow_plot.png", res=600, width=3000, height=2000)
ggplot(elbow_df, aes(x = k, y = tot_withinss)) +
  geom_line() + geom_point()+
  scale_x_continuous(breaks = 1:10) +
  labs(y = "Total within-cluster sum of squares") +
  theme_bw()
dev.off()

## 2 clusters
set.seed(1343)
k_means_cluster <- kmeans(x = Data[,Cluster_variables], centers = 2)
# Add cluster to dataset
Data$cluster <- k_means_cluster$cluster %>% as.factor()

## Mean scores of farmers in each cluster
k_means_cluster$centers
Cluster_means <- Data %>%
  group_by(cluster) %>%
  dplyr::summarise(SVODegrees = mean(SVODegrees),
                Trust_beef_farmers = mean(Trust_beef_farmers),
                Trust_dairy_farmers = mean(Trust_dairy_farmers),
                Careful_before_trust_farmers = mean(Careful_before_trust_farmers),
                Trust_farmers_met_for_first_time = mean(Trust_farmers_met_for_first_time),
                Trust_neighbours_to_control_disease = mean(Trust_neighbours_to_control_disease),
                Trust_farmers_to_control_disease = mean(Trust_farmers_to_control_disease),
                Trust_vets = mean(Trust_vets),
```

```
                Feel_respected_by_vet = mean(Feel_respected_by_vet),
                Feel_respected_by_vets = mean(Feel_respected_by_vets),
                Careful_before_trust_vets = mean(Careful_before_trust_vets),
                Trust_vet_advice_about_disease_control = mean(Trust_vet_advice_about_disease_control),
                Farmers_receive_high_quality_vet_advice = mean(Farmers_receive_high_quality_vet_advice),
                Vet_would_always_tell_truth = mean(Vet_would_always_tell_truth),
                Trust_NFU = mean(Trust_NFU),
                Feel_respected_by_NFU = mean(Feel_respected_by_NFU),
                Trust_gov_orgs = mean(Trust_gov_orgs),
                Feels_respected_by_Gov = mean(Feels_respected_by_Gov),
                Careful_before_trust_Gov = mean(Careful_before_trust_Gov),
                Trust_gov_judgements_about_disease_control = mean(Trust_gov_judgements_about_disease_control),
                Psychological_proximity_to_cows = mean(Psychological_proximity_to_cows),
                Psychological_proximity_to_beef_farmers = mean(Psychological_proximity_to_beef_farmers),
                Psychological_proximity_to_dairy_farmers = mean(Psychological_proximity_to_dairy_farmers),
                Psychological_proximity_to_neighbouring_farmers = mean(Psychological_proximity_to_neighbouring_farmers),
                Psychological_proximity_to_farming_community = mean(Psychological_proximity_to_farming_community),
                Psychological_proximity_to_vet = mean(Psychological_proximity_to_vet),
                Psychological_proximity_to_vet_community = mean(Psychological_proximity_to_vet_community),
                Psychological_proximity_to_NFU = mean(Psychological_proximity_to_NFU),
                Psychological_proximity_to_government = mean(Psychological_proximity_to_government),
                Psychological_capability = mean(Psychological_capability),
                Physical_opportunity = mean(Physical_opportunity),
                Social_opportunity = mean(Social_opportunity),
                Automatic_motivation = mean(Automatic_motivation),
                Reflective_motivation = mean(Reflective_motivation)) %>%
  as.data.frame
rownames(Cluster_means) <-  Cluster_means$cluster
Cluster_means$cluster <- NULL
Cluster_means <- Cluster_means %>%
  round(digits = 2) %>%
  t()
write.csv(Cluster_means, "Cluster_means.csv")

#### Country cluster model ####
levels(Data$Country) <- list("Wal" = "Wal", "Eng" = "Eng", "Sco" = "Sco")

Cluster_country_model <- glm(cluster ~ Country, data = Data, family = binomial)
```

```
Cluster_country_coefs <- summary(Cluster_country_model)$coefficients %>%
  as.matrix() %>%
  as.data.frame()
Cluster_country_coefs <- Cluster_country_coefs[-1,c(1,4)]
Cluster_country_coefs$Estimate <- exp(Cluster_country_coefs$Estimate)
colnames(Cluster_country_coefs) <- c("OR", "P")
Cluster_country_coefs$OR <- round(Cluster_country_coefs$OR, digits = 2)
Cluster_country_coefs$P <- round(Cluster_country_coefs$P, digits = 3)
Cluster_country_CIs <- confint(Cluster_country_model)[-1,] %>%
  exp() %>%
  round(digits = 2) %>%
  formatC(digits = 2, format = "f") %>%
  as.data.frame()
colnames(Cluster_country_CIs) <- c("low_CI", "high_CI")
Cluster_country_CIs$CI <- paste(Cluster_country_CIs$low_CI, Cluster_country_CIs$high_CI, sep = " - ")
Cluster_country_coefs <- cbind(Cluster_country_coefs, Cluster_country_CIs)
Cluster_country_coefs <- Cluster_country_coefs[,c(1:2,5)]
write.csv(Cluster_country_coefs, "Cluster_country_model.csv")

#### Country cluster model with confounders ####
levels(Data$Country) <- list("Wal" = "Wal", "Eng" = "Eng", "Sco" = "Sco")

Cluster_country_inc_conf_model <- glm(cluster ~ Country + Age + Herd_type, data = Data, family = binomial)

Cluster_country_inc_conf_coefs <- summary(Cluster_country_inc_conf_model)$coefficients %>%
  as.matrix() %>%
  as.data.frame()
Cluster_country_inc_conf_coefs <- Cluster_country_inc_conf_coefs[-1,c(1,4)]
Cluster_country_inc_conf_coefs$Estimate <- exp(Cluster_country_inc_conf_coefs$Estimate)
colnames(Cluster_country_inc_conf_coefs) <- c("OR", "P")
Cluster_country_inc_conf_coefs$OR <- round(Cluster_country_inc_conf_coefs$OR, digits = 2)
Cluster_country_inc_conf_coefs$P <- round(Cluster_country_inc_conf_coefs$P, digits = 3)
Cluster_country_inc_conf_CIs <- confint(Cluster_country_inc_conf_model)[-1,] %>%
  exp() %>%
  round(digits = 2) %>%
  formatC(digits = 2, format = "f") %>%
  as.data.frame()
```

```
colnames(Cluster_country_inc_conf_CIs) <- c("low_CI", "high_CI")
Cluster_country_inc_conf_CIs$CI <- paste(Cluster_country_inc_conf_CIs$low_CI, Cluster_country_inc_conf_CIs$high_CI, sep = " - ")
Cluster_country_inc_conf_coefs <- cbind(Cluster_country_inc_conf_coefs, Cluster_country_inc_conf_CIs)
Cluster_country_inc_conf_coefs <- Cluster_country_inc_conf_coefs[,c(1:2,5)]
write.csv(Cluster_country_inc_conf_coefs, "Cluster_country_inc_conf_model.csv")

#### Plots of variables used in clustering by cluster ####
# On same plot
Plot_data <- NULL
for(i in colnames(Data[,Cluster_variables])){
  for(j in 1:nlevels(Data$cluster)){
    x <- subset(Data, cluster==j)
    n <- nrow(x)
    mean <- mean(x[,i])
    sd <- sd(x[,i])
    margin <- qt(0.975, df = n - 1)* sd / sqrt(n)
    LCI <- mean - margin
    UCI <- mean + margin
    Plot_data <- rbind(Plot_data, c(i, j, mean, LCI, UCI))
    colnames(Plot_data) <- c("Covariate", "Cluster", "Mean", "LCI", "UCI")
  }
}
Plot_data <- as.data.frame(Plot_data)
Plot_data[,c("Covariate", "Cluster")] <- lapply(Plot_data[,c("Covariate", "Cluster")], as.factor)
Plot_data[,c("Mean", "LCI", "UCI")] <- lapply(Plot_data[,c("Mean", "LCI", "UCI")], as.numeric)
Plot_data <- Plot_data[complete.cases(Plot_data),]
ggplot(data = Plot_data, aes(y = Mean)) +
  geom_point(aes(x = Cluster)) +
  geom_errorbar(aes(ymax = UCI, ymin = LCI, x = Cluster), width = 0.25) +
  facet_grid(. ~ Covariate) +
  labs(y = "Normalised scale") +
  theme_bw()

#### Power analysis ####

# Power
Power_at_0.05 <- NULL
Power_at_0.0015 <- NULL
```

```
for(i in Dependent_variables){
  D <- Data[,c("Country",i)][complete.cases(Data[,c("Country",i)]),]
  nWal <- subset(D, Country=="Wal") %>% nrow()
  nSco <- subset(D, Country=="Sco") %>% nrow()
  nEng <- subset(D, Country=="Eng") %>% nrow()
  effect_sizeWalSco <- abs((mean(subset(D, Country=="Wal")[,i]) - mean(subset(D, Country=="Sco")[,i]))) / sd(D[,i])
  effect_sizeWalEng <- abs((mean(subset(D, Country=="Wal")[,i]) - mean(subset(D, Country=="Eng")[,i]))) / sd(D[,i])
  effect_sizeScoEng <- abs((mean(subset(D, Country=="Sco")[,i]) - mean(subset(D, Country=="Eng")[,i]))) / sd(D[,i])
  x0.05 <- pwr.t2n.test(n1 = nWal, n2 = nSco, d = effect_sizeWalSco, sig.level = 0.05)$power %>% round(digits=2)
  y0.05 <- pwr.t2n.test(n1 = nWal, n2 = nEng, d = effect_sizeWalEng, sig.level = 0.05)$power %>% round(digits=2)
  z0.05 <- pwr.t2n.test(n1 = nSco, n2 = nEng, d = effect_sizeScoEng, sig.level = 0.05)$power %>% round(digits=2)
  Power_at_0.05 <- rbind(Power_at_0.05, c(i, x0.05, y0.05, z0.05))
  x0.0015 <- pwr.t2n.test(n1 = nWal, n2 = nSco, d = effect_sizeWalSco, sig.level = 0.0015)$power %>% round(digits=2)
  y0.0015 <- pwr.t2n.test(n1 = nWal, n2 = nEng, d = effect_sizeWalEng, sig.level = 0.0015)$power %>% round(digits=2)
  z0.0015 <- pwr.t2n.test(n1 = nSco, n2 = nEng, d = effect_sizeScoEng, sig.level = 0.0015)$power %>% round(digits=2)
  Power_at_0.0015 <- rbind(Power_at_0.0015, c(i, x0.0015, y0.0015, z0.0015))
}
colnames(Power_at_0.05) <- c("Factor", "WalvSco", "WalvEng", "ScovEng")
colnames(Power_at_0.0015) <- c("Factor", "WalvSco", "WalvEng", "ScovEng")

# Save dataframes
write.csv(Power_at_0.05, "Power_analysis_0.05_sig.csv")
write.csv(Power_at_0.0015, "Power_analysis_0.0015_sig.csv")

# Effect size
Effect_size_at_0.05 <- NULL
Effect_size_at_0.0015 <- NULL
for(i in Dependent_variables){
  D <- Data[,c("Country",i)][complete.cases(Data[,c("Country",i)]),]
  nWal <- subset(D, Country=="Wal") %>% nrow()
  nSco <- subset(D, Country=="Sco") %>% nrow()
  nEng <- subset(D, Country=="Eng") %>% nrow()
  x0.05 <- (pwr.t2n.test(n1 = nWal, n2 = nSco, power = 0.8, sig.level = 0.05)$d * sd(D[,i])) %>% round(digits=2)
  y0.05 <- (pwr.t2n.test(n1 = nWal, n2 = nEng, power = 0.8, sig.level = 0.05)$d * sd(D[,i])) %>% round(digits=2)
  z0.05 <- (pwr.t2n.test(n1 = nSco, n2 = nEng, power = 0.8, sig.level = 0.05)$d * sd(D[,i])) %>% round(digits=2)
  Effect_size_at_0.05 <- rbind(Effect_size_at_0.05, c(i, x0.05, y0.05, z0.05))
  x0.0015 <- (pwr.t2n.test(n1 = nWal, n2 = nSco, power = 0.8, sig.level = 0.0015)$d * sd(D[,i])) %>% round(digits=2)
  y0.0015 <- (pwr.t2n.test(n1 = nWal, n2 = nEng, power = 0.8, sig.level = 0.0015)$d * sd(D[,i])) %>% round(digits=2)
```

```
  z0.0015 <- (pwr.t2n.test(n1 = nSco, n2 = nEng, power = 0.8, sig.level = 0.0015)$d * sd(D[,i])) %>% round(digits=2)
  Effect_size_at_0.0015 <- rbind(Effect_size_at_0.0015, c(i, x0.0015, y0.0015, z0.0015))
}
colnames(Effect_size_at_0.05) <- c("Factor", "WalvSco", "WalvEng", "ScovEng")
colnames(Effect_size_at_0.0015) <- c("Factor", "WalvSco", "WalvEng", "ScovEng")

# Save dataframes
write.csv(Effect_size_at_0.05, "Effect_size_analysis_0.05_sig.csv")
write.csv(Effect_size_at_0.0015, "Effect_size_analysis_0.0015_sig.csv")
```
